# Postoperative chronic pain following uniport vs. multiport video-assisted thoracoscopic surgery: insights from a propensity score-matched analysis

**DOI:** 10.1007/s00540-025-03624-5

**Published:** 2025-12-15

**Authors:** Yali Tian, Yiwei Zhong, Min Wang, Shujie Niu, Siyi Wang, Huaye Xu, Bingbing Li

**Affiliations:** Department of Anesthesiology, Nanjing Drum Tower Hospital, Affliated Hospital of Medical School, Nanjing University, Zhongshan Road 321, Nanjing, 210008 China

**Keywords:** Video-assisted thoracic surgery, Uniport VATS, Multiport VATS, Chronic post-surgical pain, Propensity score matching

## Abstract

**Background:**

Chronic post-surgical pain (CPSP) is a prevalent complication following video-assisted thoracic surgery (VATS), significantly affecting long-term patient outcomes. This study aimed to evaluate the influence of uniport versus multiport VATS on the incidence of CPSP and postoperative recovery at six months.

**Methods:**

Patients were stratified into uniport and multiport VATS groups, with propensity score matching (PSM). The primary endpoint was the incidence of CPSP at six months. The secondary endpoints comprise the short-term recovery outcomes within 30 days postoperatively, the quality of life at six months and group-based trajectory modeling to characterize pain trajectories over six months.

**Results:**

After PSM, 222 patients (111 per group) were included in the final analysis. A significant difference in postoperative pain intensity {3(1.8–4) vs 4(3–5), *P* = 0.007} was observed on postoperative day 3 (POD3). However, no significant difference in CPSP incidence at six months was detected between the uniport and multiport VATS groups. Multivariate logistic regression analysis of the entire cohort identified a high pain score on POD3 as an independent risk factor for CPSP development. Pain trajectory analysis revealed three distinct postoperative pain patterns over the six-month period. Patients in the high pain trajectory required more remedial analgesia and were more likely to develop CPSP.

**Conclusions:**

Uniport VATS was not associated with a lower incidence of CPSP compared to multiport VATS at six months postoperatively. A high pain score on POD3 emerged as a significant predictor of CPSP.

**Supplementary Information:**

The online version contains supplementary material available at 10.1007/s00540-025-03624-5.

## Introduction

Chronic post-surgical pain (CPSP) is a prevalent postoperative complication, with epidemiological studies reporting an incidence ranging from 10 to 50%, and approximately 10% of affected patients experiencing severe pain [1]. The International Association for the Study of Pain (IASP) defines CPSP as pain that develops or intensifies after surgery and persists for at least three months [2]. Given its potential to significantly impair patients’ quality of life and interfere with daily activities, preventing CPSP remains a critical priority [3].

Video-assisted thoracoscopic surgery (VATS) is a minimally invasive technique widely used in thoracic surgery [4]. With advancements in surgical instrumentation and techniques, VATS has evolved from conventional three-port and two-port approaches to the uniportal technique [5]. Multiport VATS provides adequate working space and facilitates optimal surgical exposure. However, traditional multiport thoracoscopy often involves larger incisions, prolonged postoperative recovery, and a higher risk of complications. In contrast, uniport VATS has gained popularity due to its advantages, including reduced surgical trauma, shorter recovery time, and potentially enhanced postoperative outcomes [6, 7].

Despite the increasing adoption of uniport VATS, the optimal surgical approach remains a subject of debate. Current literature has not conclusively established the superiority of uniport over multiport VATS in terms of clinical outcomes. Furthermore, the relationship between different VATS approaches and CPSP remains unclear. While uniport VATS is theoretically less invasive, it remains controversial whether it effectively reduces the incidence of CPSP compared to multiport techniques [8].

The primary objective of this study was to retrospectively evaluate the association between different VATS approaches and the incidence of CPSP. We hypothesized that CPSP incidence might differ between patients undergoing uniport versus multiport VATS. Additionally, we aimed to characterize distinct postoperative pain trajectories over a six-month period using group-based trajectory modeling.

## Methods

### Study design and participants

This was a single-centre, retrospective cohort study based on a prospectively collected database conducted at Nanjing Drum Tower Hospital, affiliated with Nanjing University Medical School. Ethical approval was obtained from the institutional Ethics Committee (No. 2023–286-01), and the requirement for written informed consent was waived.

We retrospectively analyzed data from adult patients (≥ 18 years) who underwent elective video-assisted thoracoscopic surgery (VATS), including lobectomy, segmentectomy, and wedge resection for pulmonary nodules, between January and December 2022. All eligible patients were followed up by telephone at 6 months postoperatively.

### Inclusion and exclusion criteria

The inclusion criteria were as follows: (1) Age ≥ 18 years; (2) American Society of Anesthesiologists (ASA) physical status I–IV. Exclusion criteria included (1) Pre-existing chronic pain conditions, severe preoperative anxiety or depression, or drug abuse; (2) History of thoracic surgery or other malignant tumours; (3) Bilateral surgery or conversion to thoracotomy; (4) Receipt of chemotherapy or radiotherapy; (5) Incomplete perioperative records; (6) Loss to follow-up or incomplete follow-up data.

### Anaesthetic management

Premedication was not routinely administered. The choice of regional nerve block technique including thoracic paravertebral block, erector spinae block, serratus anterior block and intercostal nerve block was at the discretion of the attending anaesthetist. All patients received general anesthesia. Airway management was achieved using either a laryngeal mask airway (LMA) or a double-lumen endotracheal tube, as determined by the anaesthetist. Anaesthesia was maintained with propofol, muscle relaxant and opioid based analgesia, with intermittent sufentanil as required to maintain a bispectral index (BIS) of 40–60. To prevent postoperative nausea and vomiting (PONV), 4 mg ondansetron was administered unless contraindicated. Postoperative analgesia was managed using patient-controlled intravenous analgesia (PCIA), delivering sufentanil (2 µg/kg), ondansetron (0.1 mg/kg), and dexamethasone (10 mg) in 100 mL saline at a basal infusion rate of 2 ml/h, which was continuously administered until POD 2.

### Surgical procedure

Regarding the selection criteria, we followed the following standardized process:(1) Surgeon’s technical expertise and preference: All participating surgeons in our center were proficient in both approaches. For technically mature surgeons, the choice between uniportal or multiportal access predominantly depended on their specific proficiency with the procedure and personal operative habits. (2) Tumor complexity and location: For peripheral and small-sized lesions, both approaches were generally considered appropriate, whether the procedure involved wedge resection or segmentectomy. However, in cases requiring lobectomy with systematic lymph node dissection or for centrally located tumors adjacent to major pulmonary vessels, the choice of approach was determined by the surgeon’s intraoperative assessment, prioritizing optimal exposure of the operative field and instrument maneuverability to ensure procedural safety and completeness of resection.

Uniport VATS: A 3–4 cm incision was made along the anterior to mid-axillary line at the fourth or fifth intercostal space, serving as both the observation and working port. A single chest tube was placed postoperatively. Multiport VATS: A 1–2 cm observation port was created at the mid-axillary line at the sixth or seventh intercostal space, with a separate 3 cm operative port at the anterior to mid-axillary line in the third or fourth intercostal space. Postoperative drainage was achieved through the observation port.

### Postoperative management in the ward

Upon return to the ward, multimodal analgesia was implemented in all patients. Rescue analgesia was provided with either oral tramadol(50 mg) or intravenous flurbiprofen axetil(100 mg) if the visual analogue scale (VAS) score was ≥ 4 on POD1 and POD 2. Mechanical thromboprophylaxis was applied in patients assessed as having a moderate-to-high risk of deep venous thromboembolism. Oral intake, including fluids and a light diet, was resumed 2 h postoperatively. On postoperative day 1, routine laboratory investigations—including complete blood count, coagulation profile, and serum biochemistry—were conducted, along with a standard chest radiograph. Patients were encouraged to engage in early mobilisation as part of pulmonary rehabilitation, with walking sessions of at least 6 min, three times daily. Discharge criteria included the removal of chest drains based on pleural fluid drainage less than 300 ml daily, absence of air leakage, and radiological confirmation of lung expansion. A 7-day course of oral NSAIDs analgesics was prescribed post-discharge for continued pain management.

### Data collection and follow-up

Demographic, perioperative, and postoperative data were extracted from electronic medical records and anaesthesia databases by an independent investigator. Variables included: Baseline characteristics: sex, age, ASA status, body mass index (BMI), smoking history, comorbidities, and pulmonary nodule size. Perioperative parameters: surgical approach, extent of resection, type of regional analgesia, duration of surgery, intraoperative blood loss, fluid balance, intraoperative medications, postoperative rescue analgesia, PONV, duration of chest drainage, and length of hospital stay. Assessment of pain and rescue analgesic consumption were evaluated at the bedside by another blinded investigator using the Visual Analogue Scale (VAS; 0–10) on POD1. Similarly, other postoperative outcomes including postoperative recovery, pain trajectory and persistent postsurgical pain on POD3, 5, 9, 30, 180, were assessed via telephone. The assessors were not aware whether the patient had been pre-assigned to the "single-port" or "multi-port" group. CPSP was defined as any pain at or near the surgical site with a VAS score > 0 at 6 months, after excluding alternative causes (e.g., infection, malignancy, pre-existing chronic pain). The Quality of Recovery-15 (QoR-15) questionnaire was used to evaluate global postoperative recovery [9].

### Outcomes

The primary outcome was the incidence of CPSP. The secondary outcomes included: quality of recovery (QoR-15) at 6 months, pain trajectory over 6 months, Risk factors for CPSP development, as well as acute postoperative pain intensity.

### Statistical analysis

Data were analyzed using SPSS (version 26.0) and R (version 4.3.1). Missing data were imputed using multiple imputation.

#### Descriptive and comparative analysis

Continuous variables were assessed for normality using the Shapiro–Wilk test. Normally distributed data were presented as mean ± standard deviation (SD) and compared using independent t-tests. Non-normally distributed data were reported as median (inter quartile range) and analyzed using the Mann–Whitney U test. Categorical variables were expressed as frequencies (percentages) and compared using the chi-square or Fisher’s exact test, as appropriate. To account for multiple comparisons of postoperative pain scores, a Bonferroni correction was applied, with statistical significance set at *P* < 0.05/n (where n represents the number of comparisons).

#### Propensity score matching (PSM)

To minimize baseline confounding, propensity score matching (PSM) was performed using the matchit package in R. Matching was based on sex, age, BMI, ASA classification, hypertension, diabetes, coronary artery disease, cerebral infarction, nodule size, preoperative puncture, and preoperative pain status. Nearest-neighbour matching (1:1 ratio) was performed with a caliper width of 0.05. Balance between groups was assessed using the standardised mean difference (SMD), with an SMD < 0.10 indicating adequate matching.

#### Sensitivity analysis

Inverse probability of treatment weighting (IPTW) was conducted using the IPW package in R to validate the association between surgical approach and CPSP outcomes.

#### Risk factor analysis

Multivariate logistic regression was performed to identify independent predictors of CPSP, incorporating variables with *P* < 0.05 from univariate analysis and variables with clinical significance. Results were presented as adjusted odds ratios (ORs) with 95% confidence intervals (CIs). A P value < 0.05 was considered statistically significant.

#### Pain trajectory modelling

Group-based trajectory modelling (GBTM) was used to identify distinct pain trajectories over 6 months. Models were fitted using the GBTM package in R, with the optimal number of trajectory groups selected based on Bayesian Information Criterion (BIC), Akaike Information Criterion (AIC), posterior probabilities, and class proportions. Associations between trajectory groups and clinical variables were assessed using one-way ANOVA or the Kruskal–Wallis test for continuous variables, and chi-square or Fisher’s exact test for categorical variables.

## Results

### Patient characteristics and clinical features

**Fig. 1:**
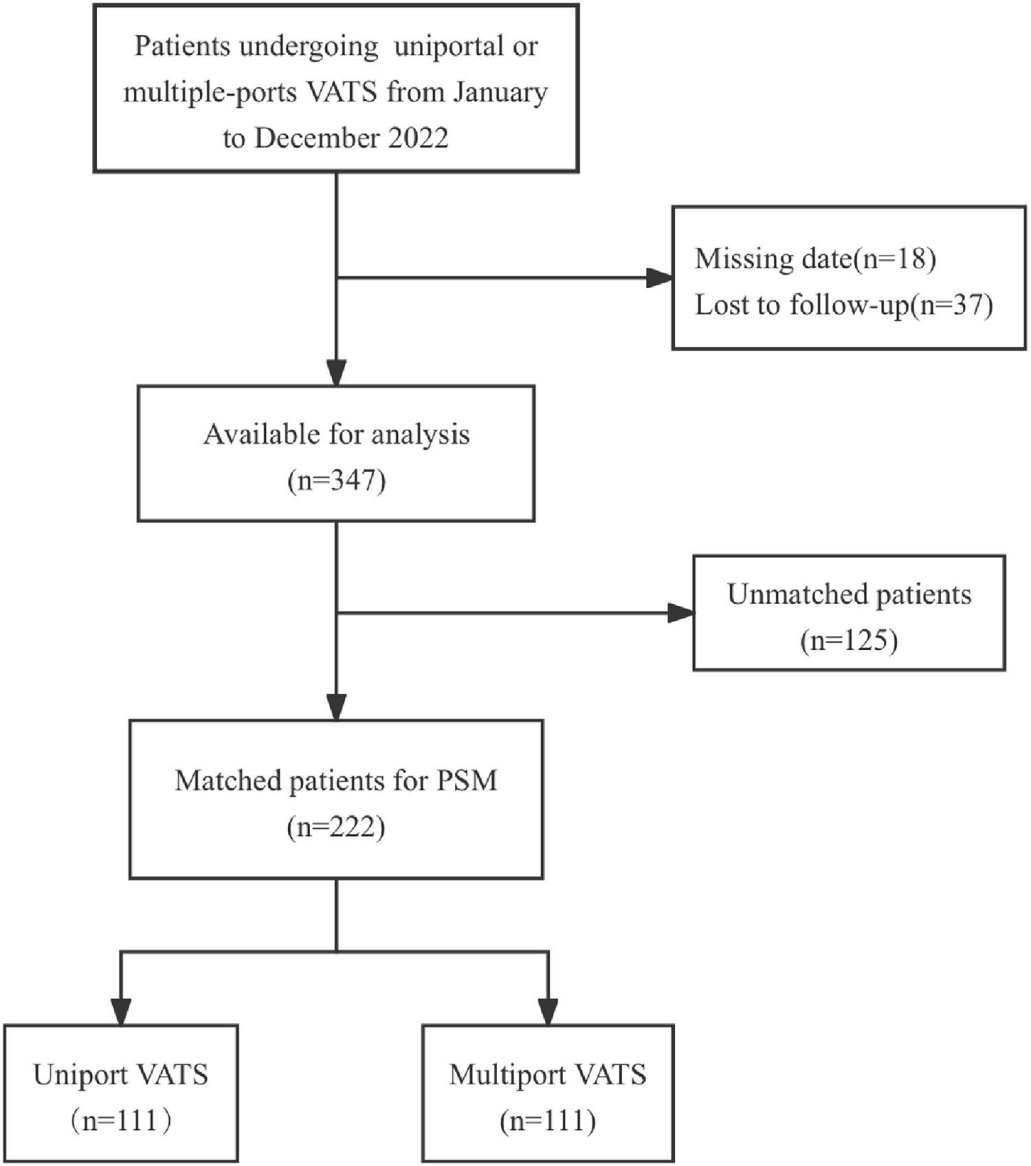
Flow diagram of case selection. VATS, Video-assisted thoracoscopic surgery; PSM, Propensity Score Matching

A total of 401 eligible patients underwent VATS between January and December 2022 were enrolled, among which 37 could not be followed up and 17 did not have complete records. Accordingly, 347 patients received follow-up, with 211 patients in uniport VATS group and 136 patients in multiport VATS group, respectively (Fig. 1).The demographic characteristics and perioperative parameters were presented in Table 1. Before matching, there were significant differences in baseline variables (SMD > 0.1). Notably, the baseline data of the two groups were balanced and comparable after PSM, with 111 patients remained in each group (Table 1). All variables showed an SMD of less than 0.1 except ASA 4.

**Table 1:** Patient characteristics for the total and propensity-matched cohorts

	Total cohort			Matched cohort		
	Uniport VATS (*n* = 211)	Multiport VATS *(n* = 136)	SMD	Uniport VATS (*n* = 111)	Multiport VATS (*n* = 111)	SMD
Age(yr)	49.2 ± 12.2	52.9 ± 12.0	−0.302	52.3 ± 12.6	51.7 ± 11.7	0.044
Gender			0.229			−0.019
Male	79(37.4)	66(48.5)		51(45.9)	50(45)	
Female	132(62.6)	70(51.5)		60(54.1))	61(55)	
BMI (kg/m^2^)	23.9 ± 3.3	24.1 ± 3.1	−0.069	24.1 ± 3.2	23.8 ± 3.1	0.095
ASA physical status						
I	0(0)	1(0.7)	−0.119	0(0)	0(0)	0.000
II	66(31.3)	46(33.8)	−0.053	38(34.2)	35(31.5)	0.058
III	141(66.8)	89(65.4)	0.030	72(64.9)	46(68.5)	−0.076
IV	4(1.9)	0(0)	0.197	1(0.9)	0(0)	0.135
Smoking status	14(6.6)	10(7.4)	−0.029	7(6.3)	8(7.2)	−0.036
Hypertension	37(17.5)	29(21.3)	−0.099	24(21.6)	22(19.8))	0.047
Diabetes	10(4.7)	8(5.9)	−0.054	5(4.5)	7(6.3)	−0.085
Coronary heart disease	2(0.9)	1(0.7)	0.022	1(0.9)	1(0.9)	0.000
Cerebral infarction	2(0.9)	3(2.2)	−0.130	2(1.8)	2(1.8)	0.000
Preoperative puncture	128(60.7)	88(64.7)	−0.083	71(64)	75(67.6)	−0.074
Nodule size(mm)	10(8–13)	11(9–16)	−0.379	11(8–15)	10(9–14)	−0.052
Preoperative hemameba(10^9/L)	5.9(4.7–6.8)	5.75(4.7–7.23)	−0.184	6.1(4.9–6.7)	6(4.9–7)	0.015
Preoperative CRP(mg/L)	2(1.7–2.6)	1.9(1.38–2.23)	−0.050	2.1(1.7–2.9)	1.9(1.6–2.6)	0.030
Preoperative albumin(g/L)	44.3 ± 1.7	44.0 ± 2.0	0.147	44.2 ± 1.7	44.2 ± 1.8	−0.015
Preoperative hemoglobin(g/L)	142(134–156)	144(133.8–155.3)	0.005	144.1 ± 17.5	144.8 ± 13.4	−0.016

Data are presented as *n* (%), mean ± standard deviation, or median (interquartile range). VATS, Video-assisted thoracoscopic surgery; BMI, Body mass index; ASA, American Society of Anesthesiologists; CRP, C-reactive protein

A comparison of the postoperative data of patients for the propensity matched cohorts was presented in Table S1. No difference was detected between the two groups.

### Primary outcome

#### Acute and chronic pain characteristics

Out of the 347 individuals in the study, 86(24.8%) developed chronic pain at 6 months before matching. As Table 2 shows, the incidence of CPSP was 27% (*n* = 30) and 26.1% (*n* = 29) in the uniport VATS and multiport VATS groups, respectively, at 6 months following the operation. However, the significance of the difference was not substantial(*P* = 0.879, OR = 1.047, 95%CI 0.57–1.9). Regarding the characteristics of CPSP, the subjective symptoms of prickling pain(14.7%), burning pain(3%), shooting pain(1%), numbness(7%), colding pain(8%) and formication pain(1%).

**Table 2:** Pain severity and chronic pain on postoperative days based on PSM and IPTW

	After PSM				After IPTW			
	Uniport VATS (n = 111)	Multiport VATS (n = 111)	Z/χ^2^	*P*	Uniport VATS (n = 211)	Multiport VATS (n = 136)	Z/χ^2^	*P*
*Primary outcome*								
Incidence of CPSP on POD180	30(27)	29(26.1)	0.023	0.879	50(23.7)	36(26.7)	0.389	0.533
*Secondary outcomes*								
POD1VAS	3(3–3)	3(2–3)	−0.189	0.85	3(2–3)	3(2–3)	−0.842	0.400
POD3VAS	3(1.8–4)	4(3–5)	−2.717	**0.007**	3(2–4)	4(3–5)	−3.048	**0.002**
POD5VAS	3(2–3)	3(2–3.3)	−0.331	0.741	3(2–3)	3(2–3)	−1.087	0.277
POD9VAS	2(1–3)	2(1.8–3)	−1.091	0.275	2(1–3)	2(1–3)	−1.166	0.244
POD30VAS	1(0–2)	0.5(0–1.3)	−0.324	0.746	1(0–2)	0(0–1)	−0.451	0.652
POD180VAS	0(0–0)	0(0–1.3)	−0.222	0.824	0(0–0)	0(0–1.5)	−0.54	0.589

Data are presented as *n* (%) or median (interquartile range). PSM, Propensity Score Matching; IPTW, Inverse Probability of Treatment Weighting; VATS, Video-assisted thoracoscopic surgery; CPSP, Chronic Post-Surgical Pain; VAS, Visual Analogue Scale

Bold values represent statistical significance at *P* < 0.05

The overall VAS score of patients in terms of postoperative acute pain from the first postoperative day to 30 days is plotted in Table 2. The VAS score of acute postoperative pain at POD3 was significantly reduced in uniport VATS group comparing to multiport VATS group{3(1.8–4) vs 4(3–5), *P* = 0.007}. Whereas, there was no statistical difference observed between the scores of the two groups on POD 1, 5, 9, 30.

#### Sensitivity analyses

In the sensitivity analysis, characteristics except ASA4 were adequately balanced after IPTW in the two groups with an SMD of less than 0.1(figure S1). The primary outcomes showed in Table 2 were consistent with the preceding one.

Subsequently, a logistic regression analysis on overall patients was used to further examine the incidence of CPSP, considering the similar incidence of CPSP between the matched groups. Whereas, only pain score on POD3 was shown to be the risk factor of chronic pain at 6 months following surgery according to the multivariate regression analysis (OR 1.42, 95% CI 1.12–1.79; *P* = 0.003; Table 3). The Hosmer–Lemeshow test indicated that the model showed no evidence of a lack of fit (*P* = 0.247). Figure S2 shows the results of different models on the association between uniportal port VATS and the risk of CPSP.

**Table 3:** Multivariate logistic regression analysis for the development of CPSP in the total cohort within six months

	B	Wald	*P*	OR	95%CI
Age	−0.009	0.716	0.397	0.99	0.97–1.01
Gender	−0.201	0.605	0.437	0.818	0.49–1.36
Surgical time	0.001	0.024	0.877	1.00	0.99–1.00
Extent of excision	−0.665	1.917	0.166	0.51	0.20–1.32
Ports	−0.103	0.096	0.757	0.90	0.47–1.73
Drainage on the first postoperative day	−0.003	2.28	0.131	0.99	0.99–1.00
VAS on POD 3	0.35	8.64	**0.003**	1.42	1.12–1.79
Cough on POD30	−0.457	1.257	0.262	0.63	0.29–1.41

CPSP, Chronic Post-Surgical Pain; OR, Odds ratio; CI, Confidence interval; VAS, Visual Analogue Scale

Bold values represent statistical significance at *P* < 0.05

### Secondary outcomes

#### Other follow-up data for patients

We observed a difference in coughing between the two groups on POD30(Table S2). But after Bonferroni correction, the difference was not statistically significant. Therefore, the difference in postoperative coughing between the two groups is not statistically significant. Additionally, the quality of life(*P* = 0.831) did not differ significantly between the matched groups as measured by total scores on QOR15(Table S2).

#### The trajectory of pain within 6 months

**Fig. 2:**
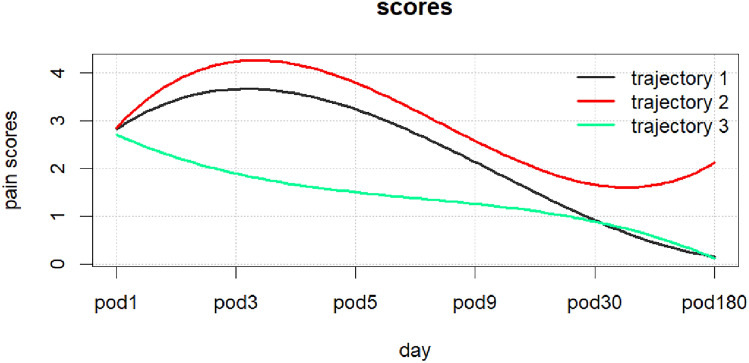
The trajectory of pain for the total cohorts during the 6 months recovery

Table S3 presented model fitting for group-based trajectory analysis. Although trajectory 4 has the lowest BIC and AIC values, its class proportions are slightly inferior, and considering professional explainability, trajectory 3 was ultimately selected. The best-fitting model included three trajectory of pain(low, moderate and high), which represented graphically in Fig. 2. In total, 35.2% (122 of 347) were in the low pain group. 41.5% (144 of 347) were in the moderate pain group. 23.3% (81 of 347) were in the high pain group. For this entire sample, the daily pain for the first day after surgery was similar. Maximal pain intensity was greater in the high trajectory group compared with the other trajectory groups from POD 3 afterward. Subsequently, we examined the relationship between several preoperative factors and these trajectory groups. Table 4 summarized patient characteristics by trajectory group. However, the trajectory group was not statistically significantly associated with the variables presented except smoking history(*P* = 0.035). We combined patients in the low pain and moderate pain trajectory groups and compared with those in the high pain trajectory group. The results showed that the proportion of remedial analgesia was higher(*P* = 0.01) but the proportion of smoking patients was lower(*P* = 0.02) in the high pain trajectory group and it was more likely to develop into chronic pain.

**Table 4:** Patient Demographics by Classes

Patient Demographics	Low (*n* = 122)	Moderate (*n* = 144)	High (*n* = 81)	*P* value
Age(yr)	49.7 ± 12.3	52.1 ± 12.1	49.4 ± 12.4	0.161
Sex				0.180
Male	58(47.5)	59(41)	28(34.6)	
Female	64(52.5)	85(59)	53(65.4)	
BMI (kg/m^2^)	24.2 ± 3.4	24.1 ± 3.2	23.4 ± 3.0	0.254
ASA physical status				0.408
I	0(0)	0(0)	1(1.2)	
II	45(36.9)	41(28.5)	26(32.1)	
III	75(61.5)	102(70.8)	53(65.5)	
IV	2(1.6)	1(0.7)	1(1.2)	
Smoking status	13(10.7)	10(6.9)	1(1.2)	**0.035**
Hypertension	21(17.2)	29(20.1)	16(19.8)	0.817
Diabetes	3(2.5)	10(6.9)	5(6.2)	0.233
Coronary heart disease	1(0.8)	2(1.4)	0(0)	0.557
Cerebral infarction	1(0.8)	1(0.7)	3(3.8)	0.148
Preoperative puncture	77(63.1)	87(60.4)	52(64.2)	0.829
Nodule size(mm)	11(8–14)	10(8–15)	10(8–13)	0.419
Preoperative hemameba(10^9/L)	5.7(4.6–7.0)	5.7(4.9–6.8)	6.1(4.8–7.2)	0.686
Preoperative CRP(mg/L)	2.1(1.8–2.6)	1.9(1.7–2.5)	1.9(1.4–2.3)	0.183
Preoperative albumin(g/L)	44.3 ± 2.0	43.9 ± 1.8	44.4 ± 1.6	0.163
Preoperative hemoglobin(g/L)	144(134–157)	143(133–154.5)	142(137–154)	0.638
uniportal port	80(65.6)	80(55.6)	51(63)	0.225
Nerve Block	113(92.6)	136(94.5)	75(92.6)	0.81
Dexmedetomidine	105(86.1)	122(84.7)	65(80.2)	0.523
Remifentanil dosage(mg)	0.5(0.3–0.6)	0.5(0.4–0.7)	0.5(0.3–0.7)	0.301
crystal(ml)	500(500–1000)	500(500–1000)	500(500–1000)	0.189
colloid(ml)	500(500–500)	500(500–500)	500(500–500)	0.478
Bleeding(ml)	50(20–50)	50(10–50)	30(10–50)	0.891
Urine(ml)	300(100–400)	300(175–400)	275(100–400)	0.916
Extent of excision				0.777
Wedge resection	71(58.2)	77(53.5)	49(60.6)	
Segmentectomy	30(24.6)	37(25.7)	16(19.8)	
Lobectomy	21(17.2)	30(20.8)	16(19.8)	
Surgical time(min)	70(50–86.25)	70(50–95)	65(50–90)	0.773
PONV	22(18.0)	22(15.3)	10(12.3)	0.545
Use of rescue	24(19.7)	35(24.3)	28(34.6)	0.059

Patient Demographics	Low (*n* = 122)	Moderate (*n* = 144)	High (*n* = 81)	*P* value
Length of hospital stay(d)	2(2–2)	2(2–2)	2(2–2)	0.327
Drainage tube removal time(d)	2(2–2)	2(2–2)	2(2–2)	0.534
Drainage on postoperative day(ml)	50(10–100)	50(10–100)	35(10–95)	0.435
Drainage on the first postoperative day(ml)	120(50–210)	100(50–200)	100(50–150)	0.214
Postoperative hemameba (10^9/L)	12.2 ± 3.2	12.4 ± 3.7	12.9 ± 3.0	0.415
Postoperative CRP(mg/L)	29.6(17.9–46.4)	29.9(15.5–26.3)	30.9(17.3–52.2)	0.234
Postoperative albumin(g/L)	39.6 ± 2.1	39.2 ± 1.9	39.3 ± 2.0	0.296
Postoperative hemoglobin(g/L)	132.2 ± 16.6	131.5 ± 15.1	131.3 ± 12.0	0.95

Data are presented as* n* (%), mean ± standard deviation or median (interquartile range). BMI, Body mass index; ASA, American Society of Anesthesiologists; PONV, Postoperative Nausea and Vomiting; CRP, C-reactive protein

Bold values represent statistical significance at *P* < 0.05

## Discussion

In recent years, uniport VATS has been increasingly adopted across multiple centers, demonstrating advantages over conventional two-port or three-port techniques in reducing surgical trauma and improving early postoperative recovery. However, the incidence of chronic post-surgical pain (CPSP) remains a concern, and limited research has specifically evaluated whether uniport VATS confers a significant advantage in mitigating CPSP. The primary finding of this retrospective study was that the prevalence of CPSP at 6 months did not significantly differ between the uniport and multiport VATS groups. However, pain intensity on postoperative day 3 (POD3) was significantly higher in the multiport VATS group. Furthermore, multivariate analysis identified higher pain scores on POD3 as an independent risk factor for CPSP development at 6 months.

The reported incidence of CPSP following VATS varies widely, ranging from 7.7% to 50% in previous studies, likely due to differences in diagnostic criteria, perioperative analgesic strategies, and follow-up durations [10, 11]. In our cohort, the overall CPSP incidence was 24.78% at 6 months. Consistent with our findings, a prospective study by Liu and his colleagues [12] found no significant difference in pain scores at 6 months between uniport and two-port VATS. Conversely, another prospective study suggested a lower CPSP incidence with uniport VATS [13]. A recent meta-analysis also concluded that postoperative VAS scores were comparable between uniport and multiport VATS, highlighting the inconclusive impact of uniportal techniques on CPSP. [14] The variability in findings may be attributed to differences in individual pain thresholds, preoperative pain conditions, and institutional pain management protocols, which could obscure potential benefits associated with uniport VATS [15].

Acute postoperative pain is a well-established predictor of CPSP. [16] In our study, higher pain scores on POD3 were associated with an increased risk of CPSP, aligning with findings from Bayma and colleagues [17], who reported that greater pain severity within the first 3 postoperative days was predictive of CPSP, with an area under the curve of 0.73. The transition from acute to chronic pain is thought to involve structural and functional remodeling within the nervous system, including synaptic reorganization and central sensitization, leading to persistent pain despite tissue healing [18, 19]. Furthermore, pain score on POD3 was linearly correlated with CPSP (*P* = 0.914) confirmed by BOX-Tidwell test in our study. Our study confirmed that the pain score on POD3 served as a strong and practical clinical predictor for CPSP. The key clinical implication of this finding lied in providing a simple and accessible window for early identification of high-risk CPSP patients. For patients with elevated pain scores on POD 3, clinicians should regard this as a ‘warning sign’ and consider initiating a multimodal analgesia protocol. Based on relevant literature [20, 21], a prospective intervention strategy may include: 1) Systemic analgesia is essential and the first step to relieve the patient, such as non-steroidal anti-inflammatory drugs (NSAIDs), gabapentin, Ketamine and opioids; 2) Regional analgesia must be performed in addition to systemic multimodal analgesia, such as epidural and paravertebral blocks, which were the two most established techniques; 3) Non-drug techniques, such as transcutaneous electrical nerve stimulation (TENS). Consequently, future research should focus on developing and validating early intervention protocols based on risk stratification, thereby transforming the prediction of CPSP into effective prevention.

Trajectory analysis was a powerful tool for evaluating dynamic postoperative pain patterns. To the best of our knowledge, many early studies on postoperative pain assessed pain intensity on a single day after surgery or the initial postoperative days. [22] The current investigation applied trajectory analysis to delineate patterns of postoperative pain over a 6-month recovery period in patients undergoing Video-Assisted Thoracoscopic Surgery (VATS). This approach sought to move beyond simplistic pain assessments and capture the dynamic nature of pain resolution or persistence in this specific surgical population. The results of this study, particularly the delineation of three divergent pain trajectories over a 6-month period, robustly underscore the heterogeneous nature of the postoperative pain experience, even following a relatively standardized minimally invasive procedure such as VATS [23]. Our study found that patients in the high pain trajectory group required more remedial analgesia. In addition, a critical finding of this study was the strong association between membership in the high pain trajectory (Trajectory 2) and an increased likelihood of developing Chronic Post-Surgical Pain (CPSP). In contrast, patients in the moderate-resolving (Trajectory 1) and low-resolving (Trajectory 3) pain groups exhibited a more favorable course, with a gradual decline in pain intensity over time and a lower incidence of CPSP. The current study’s observation that patients in the high pain trajectory were more likely to develop CPSP resonates with Althaus et al.’s conclusion that their problematic group experienced significantly greater pain persistence^.^ [24] Research by Vasilopoulos and colleagues has also significantly contributed to the understanding of postoperative pain trajectories [23]. Their studies have identified multiple distinct postoperative pain intensity trajectories which, importantly, were often defined by specific patient-related factors, such as younger age, female sex, higher levels of preoperative anxiety, and more pronounced preoperative pain behaviors have been associated with an increased likelihood of belonging to a higher or more problematic pain trajectory in their cohorts. In stark contrast, the present study, focusing on a VATS cohort, "did not find key differentiators of trajectory group assignments for postoperative pain." This divergence warrants careful consideration. Firstly, the current study may not have encompassed the same breadth or depth of patient-related variables as those measured by Vasilopoulos and colleagues. Secondly, patients undergoing elective VATS procedures might represent a more homogeneous group in terms of certain baseline characteristics compared to the more diverse populations often included in studies of general or mixed surgical types. This relative homogeneity could make it more challenging to isolate specific patient factors that differentiate trajectory membership. The clear delineation of these varying recovery pathways emphasizes the pressing need for a shift away from standardized, one-size-fits-all approaches towards more individualized pain management strategies. Understanding these dynamic pain patterns can inform the development of tailored interventions, potentially initiated early in the postoperative course for patients trending towards unfavorable trajectories. The fundamental purpose of classifying patients into distinct pain trajectories was to achieve "precision analgesia" and "preventive analgesia," rather than passively responding to pain [25]. Our study found that the proportion of patients requiring rescue analgesia was significantly higher in the ‘high pain trajectory’ group compared to the mild-to-moderate pain trajectory groups. This result not only validated the clinical validity of the trajectory classification but, more importantly, provides a clear intervention target for implementing individualized perioperative analgesia. Our findings strongly suggested that for high-risk patients identified as belonging to the ‘high pain trajectory,’ a preventive analgesic strategy should be adopted. This includes the combined application of regional nerve blocks and non-opioid adjuvant medications (such as non-steroidal anti-inflammatory drugs (NSAIDs), gabapentin, ketamine) preoperatively or intraoperatively, coupled with a more proactive analgesic regimen in the immediate postoperative period. During the postoperative period, a fixed and safe background infusion or scheduled administration regimen of opioids should be established for these patients, rather than relying solely on patient complaints [21]. Additionally, more frequent pain assessments (e.g., every 1–2 h) are necessary for this patient population to enable prompt intervention as soon as possible, instead of waiting until the pain becomes unbearable. The aim is to reduce the need for rescue analgesia, improving the patient experience, and potentially lowering the risk of chronic pain development. Conversely, for patients in mild-to-moderate pain trajectory groups, a simplified analgesic protocol can be employed to maximize benefits and minimize drug-related adverse effects. Consequently, the core clinical value of postoperative pain trajectory classification lies in its ability to achieve precise risk stratification. Future research should prioritize the identification of reliable predictors for these trajectories within VATS populations and the evaluation of targeted interventions aimed at optimizing pain relief and reducing the incidence of chronic pain. Ultimately, leveraging pain trajectory analysis holds considerable promise for improving patient outcomes and enhancing the quality of recovery after VATS.

An unexpected finding in our study was the inverse relationship between smoking history and postoperative pain intensity, with a lower proportion of smokers in the high pain trajectory group. Similar observations have been reported in previous studies. Chen and colleagues [11] found that smokers undergoing VATS had a lower risk of developing CPSP, suggesting potential sex-related differences in pain perception. However, other studies have implicated smoking in heightened pain sensitivity via inflammatory and oxidative stress pathways [26]. Sun and colleagues [27] identified smoking history as an independent risk factor for acute postoperative pain. While our findings do not suggest that smoking is irrelevant to pain intensity, they indicate that smoking status may not be a primary determinant in postoperative pain trajectory classification [23].

This study has several limitations. First, its retrospective, single-centre design may introduce selection bias and limit generalizability. Although propensity score matching (PSM) was employed to mitigate confounding, residual confounders cannot be entirely excluded. Second, preoperative anxiety and depression, which may influence pain perception, were not comprehensively assessed; we relied on self-reported patient histories rather than validated psychological screening tools. However, previous studies suggest that preoperative psychosocial factors may not be strongly associated with CPSP. [17] Third, we did not specifically evaluate neuropathic pain, which, although considered a minor component of CPSP after VATS, remains an important aspect warranting further investigation [28]. Fourth, the specific dosages of various analgesic medications were not captured during the data collection process, which may have a more insightful interpretation of the results. In this study, PCIA utilization was maintained until POD 2 in both patient cohorts. Meanwhile, the comparable requirements for rescue analgesia between the propensity score-matched groups suggest similar overall analgesic demands, thereby reducing concerns about substantial confounding effects on our primary pain outcomes.

## Conclusions

Our findings suggest that, compared with multiport VATS, uniport VATS does not confer a significant advantage in reducing CPSP incidence at 6 months postoperatively. Acute postoperative pain, particularly on POD3, plays a critical role in CPSP development. Additionally, trajectory analysis revealed three distinct pain patterns, emphasizing the heterogeneous nature of postoperative pain and the need for tailored pain management approaches. Future prospective studies are required to confirm these findings and explore potential interventions to mitigate CPSP risk.

## Supplementary Information

Below is the link to the electronic supplementary material.

Supplementary file1 (DOCX 260 KB)

## Data Availability

The datasets used andanalysed during the current study available from the corresponding author on reasonable request.
