# Supplementary material for: Postoperative chronic pain following uniport vs. multiport video-assisted thoracoscopic surgery: insights from a propensity score-matched analysis
Source: J Anesth. 2025 Dec 15;40(4):501–11. doi: 10.1007/s00540-025-03624-5 (PMC13427939; doi:10.1007/s00540-025-03624-5)
Supplement: Supplementary file 1 — Supplementary file1 (DOCX 260 KB) [file 540_2025_3624_MOESM1_ESM.docx]

Table S1 Postoperative data of patients for the propensity matched cohorts.

|  | Uniport VATS（*n*=111） | Multiport VATS（*n*=111） | t/Z/χ^2^ | *P* |
| --- | --- | --- | --- | --- |
| Remifentanil dosage(mg) | 0.5(0.3 - 0.6) | 0.5(0.4 - 0.7) | -1.377 | 0.169 |
| Sufentanil dosage(mg) | 0.05（0.04，0.05） | 0.05（0.04，0.05） | -0.636 | 0.525 |
| crystal(ml) | 500(500 -1000) | 500(500 -1000) | -1.451 | 0.147 |
| colloid(ml) | 500(500 - 500) | 500(500 - 500) | -0.089 | 0.929 |
| Bleeding(ml) | 50(10 - 50) | 50(20 - 50) | -0.819 | 0.413 |
| Urine(ml) | 300(150 - 400) | 300(150 - 400) | -0.422 | 0.673 |
| Nerve Block  None  Paravertebral  Erector spinal  Others | 10(8.5)  65(55.6)  32(27.4)  10(8.5) | 4(3.4)  66(56.4)  42(35.9)  5(4.3) | 5.597 | 0.133 |
| Extent of excision  Wedge resrction  Segmentectomy  Lobectomy | 66(56.4)  27(23.1)  24(20.5) | 70(59.8)  28(23.9)  19(16.2) | 0.717 | 0.699 |
| Surgical time(min) | 70(50 - 90) | 70(50 - 90) | -0.428 | 0.668 |
| PONV | 17(14.5) | 27(13.7) | 0.035 | 0.851 |
| Use of rescue | 29(26.1) | 20(18) | 0.094 | 0.759 |
| Frequency of remedial analgesia  Once  Twice | 25(22.5)  4(3.6) | 17(15.3)  3(2.7) | 1.492 | 0.474 |
| First request of rescue(h) | 0(0 - 0) | 0(0 - 0) | -0.946 | 0.344 |
| Length of hospital stay(d) | 2(2 - 2) | 2(2 - 2) | -1.265 | 0.206 |
| Drainage tube removal time(d) | 2(2 - 2) | 2(2 - 2) | -1.524 | 0.128 |
| Drainage on postoperative day(ml) | 50(10 - 110) | 50(10 - 100) | -0.007 | 0.994 |
| Drainage on the first postoperative day(ml) | 110(100 - 200) | 100(50 - 200) | -2.005 | 0.050 |
| Postoperative hemameba(10^9 L^-1^) | 12.6±3.3 | 12.6±3.6 | 0.029 | 0.977 |
| Postoperative CRP(mg L^-1^) | 29.5(17.4 - 44.7) | 28.2(16.7 - 46.8) | -0.051 | 0.959 |
| Postoperative albumin(g L^-1^) | 39.2±1.9 | 39.5±2.0 | 1.004 | 0.317 |
| Postoperative hemoglobin(g L^-1^) | 131.9±16.2 | 133.7±14.2 | 0.887 | 0.376 |

Data are presented as *n* (%), mean ± standard deviation or median (interquartile range). VATS Video-assisted thoracoscopic surgery; PONV Postoperative Nausea and Vomiting.

Table S2 Follow-up data for patients within six months.

|  | Uniport VATS（*n*=111） | Multiport VATS（*n*=111） | Z/χ^2^ | *P* |
| --- | --- | --- | --- | --- |
| Cough on POD3 | 64(57.7) | 71(64.0) | 0.781 | 0.377 |
| Cough on POD5 | 66(59.5) | 80(72.1) | 0.907 | 0.341 |
| Cough on POD9 | 49(44.1) | 57(51.4) | 0.7 | 0.403 |
| Cough on POD30 | 31(27.9) | 15(13.5) | 7.534 | **0.020** |
| Quality Of Recovery on POD180 | 80(70 - 90) | 82.5(73.8 - 90) | -0.214 | 0.831 |

Data are presented as *n* (%) or median (interquartile range).

Bold values represent statistical significance at P<0.05.

Table S3 Trajectory Model Fitting.

| Fit statistic | Number of classes | | | | |
| --- | --- | --- | --- | --- | --- |
|  | 1 | 2 | 3 | 4 | 5 |
| BIC | 6977.148524 | 6665.583656 | 6558.716493 | 6554.236298 | 6557.010077 |
| AIC | 6948.943103 | 6609.172813 | 6462.818061 | 6424.49136 | 6393.418634 |
| APPA |  | class 1, 0.91 | class 1, 0.85 | class 1, 0.83 | class 1, 0.84 |
|  |  | class 2, 0.90 | class 2, 0.86 | class 2, 0.91 | class 2, 0.92 |
|  |  |  | class 3, 0.91 | class 3, 0.78 | class 3, 0.78 |
|  |  |  |  | class 4, 0.88 | class 4, 0.89 |
|  |  |  |  |  | class 5, 0.81 |
| class proportion | class 1,1 | class 1, 0.461 | class 1, 0.396 | class 1, 0.404 | class 1, 0.3153 |
|  |  | class 2,0.539 | class 2, 0.252 | class 2, 0.0919 | class 2, 0.0503 |
|  |  |  | class 3, 0.352 | class 3, 0.1884 | class 3, 0.2162 |
|  |  |  |  | class 4, 0.3157 | class 4, 0.3345 |
|  |  |  |  |  | class 5, 0.0836 |

BIC, Bayesian Information Criterion; AIC, Akaike information criterion; APPA, Average Posterior Probability of Assignment

Figure S1. The distribution of standardized mean differences of covariates in the

unmatched and matched samples based on IPTW. **
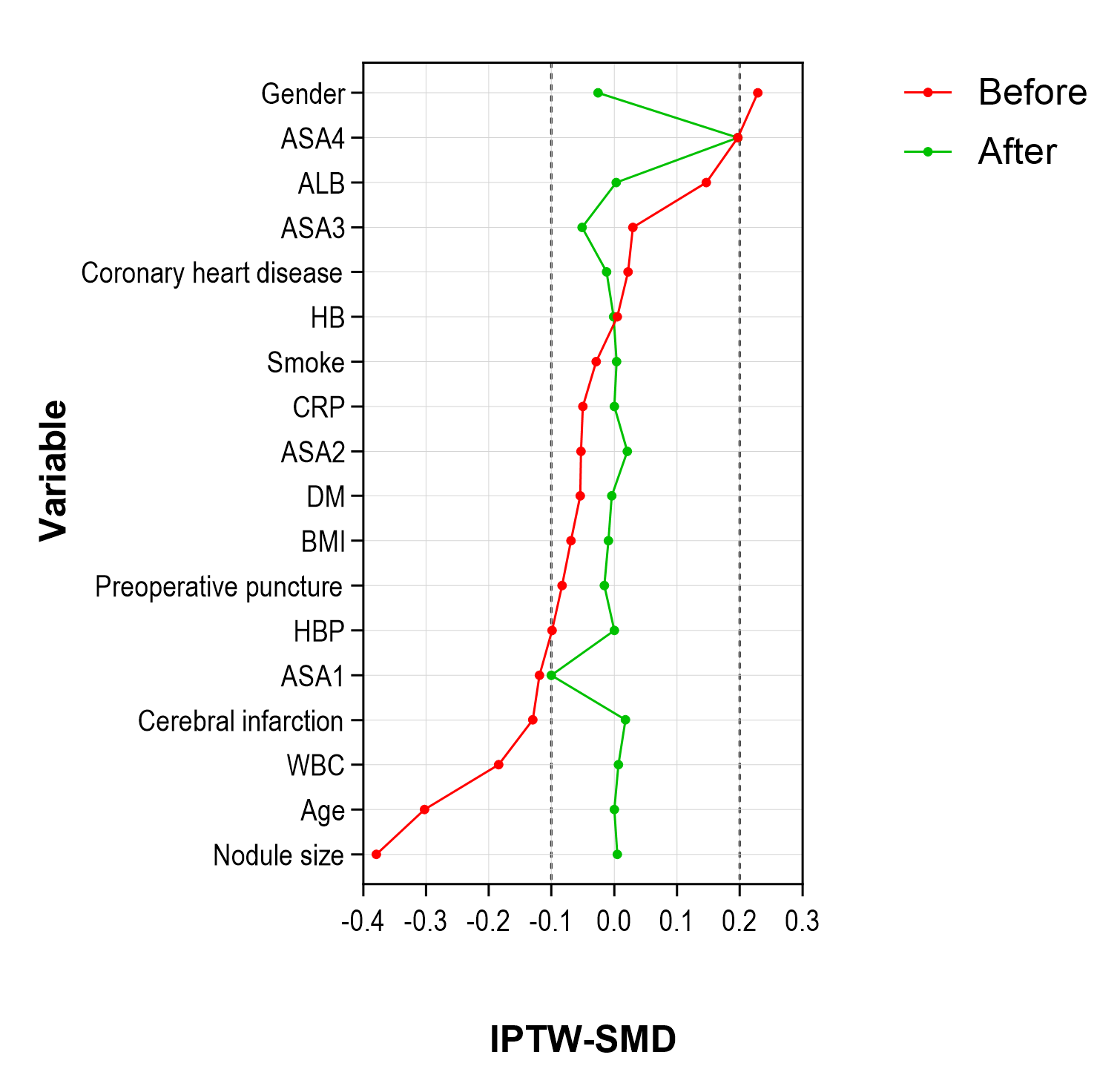
**

ASA American Society of Anesthesiologists; BMI Body mass index; IPTW Inverse Probability of Treatment Weighting; SMD Standardized mean differences.

Figure S2 Results of different models on the association between uniportal port VATS and the risk of CPSP. **
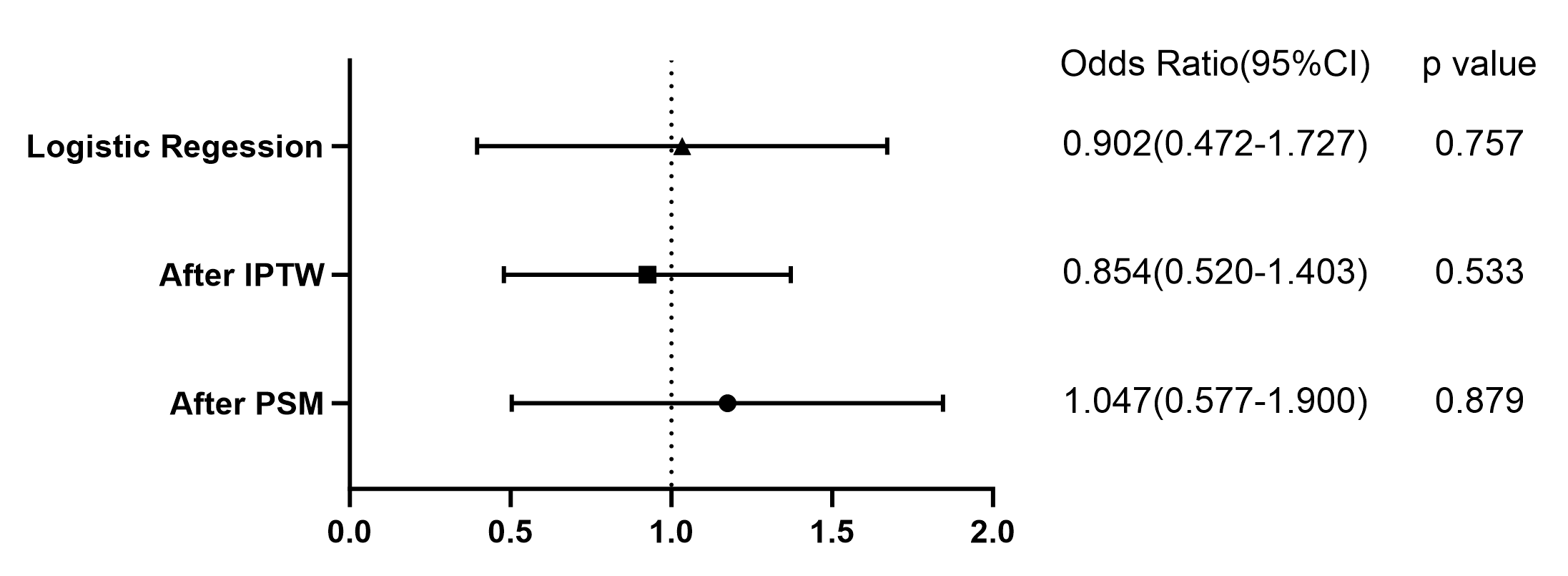
**

PSM, Propensity Score Matching; IPTW, Inverse Probability of Treatment Weighting; CI, confidence interval.
